# Endoscopic Diagnosis and Treatment of a Case of Respiratory Papillomatosis

**DOI:** 10.1155/DTE.3.183

**Published:** 1997

**Authors:** T. Hirano, C. Konaka, S. Okada, H. Shibanuma, E. Shimizu, T. Okunaka, H. Sakai, Y. Ebihara, H. Kato

**Affiliations:** 1 Department of Surgery Tokyo Medical Colleg 6-7-1 Nishishinjuku Shinjuku-ku Tokyo 160 Japan; 2 Department of Pathology Tokyo Medical Colleg 6-7-1 Nishishinjuku Shinjuku-ku Tokyo 160 Japan

## Abstract

Respiratory papilloma is a rare lesion that arises in the larynx, trachea and bronchus. We describe a patient with laryngeal papilloma that spread to the trachea and which was effectively treated by Nd-YAG laser. Two years after the initial treatments of the laryngeal and tracheal papillomas, a recurrent lesion (solitary papilloma) was observed on the membranous portion of the trachea. We examined the recurrent lesion by bronchoscopy including bronchoscopic ultrasound (US), helical computed tomography (CT) and tracheal biopsies. Respiratory papillomatosis sometimes shows either malignant transformation or invasion to tracheal wall without displaying cytohistological atypia. Therefore, we concluded that bronchoscopic US and helical CT were useful for deciding on therapeutic strategy in cases of recurrence of tracheal papilloma.


*Diagnostic and Therapeutic Endoscopy*, 1997, Vol. 3, pp. 183–187
Reprints available directly from the publisher
Photocopying permitted by license only

© 1997 OPA (Overseas Publishers Association)
Amsterdam B.V. Published in The Netherlands
by Harwood Academic Publishers
Printed in Singapore

### Endoscopic Diagnosis and Treatment of a Case of Respiratory Papillomatosis

T. HIRANO[1], C. KONAKA[1], S. OKADA[1,2], H. SHIBANUMA[1], E. SHIMIZU[1], T. OKUNAKA[1], H. SAKAI[1], Y. EBIHARA[2] and H. KATO[1]

*Department of Surgery[1] and Department of Pathology[2], Tokyo Medical College, 6-7-1 Nishishinjuku, Shinjuku-ku, Tokyo 160, Japan*

*(Received 8 July 1996; in final form 27 September 1996)*

**Respiratory papilloma is a rare lesion that arises in the larynx, trachea and bronchus. We describe a patient with laryngeal papilloma that spread to the trachea and which was effectively treated by Nd-YAG laser. Two years after the initial treatments of the laryngeal and tracheal papillomas, a recurrent lesion (solitary papilloma) was observed on the membranous portion of the trachea. We examined the recurrent lesion by bronchoscopy including bronchoscopic ultrasound (US), helical computed tomography (CT) and tracheal biopsies. Respiratory papillomatosis sometimes shows either malignant transformation or invasion to tracheal wall without displaying cytohistological atypia. Therefore, we concluded that bronchoscopic US and helical CT were useful for deciding on therapeutic strategy in cases of recurrence of tracheal papilloma.**

*Keywords:* Respiratory papillomatosis, bronchoscopy, bronchoscopic ultrasound, bronchoscopic vaporization

### INTRODUCTION

Respiratory papillomatosis is an uncommon entity which can sometimes obstruct the tracheobronchial tract. The disease is characterized by multiple growths from the larynx to the bronchi, recurrence and association with human papilloma virus (particularly types 6 and 11)[1–3]. Respiratory papilomatosis has been divided into juvenile onset type and adult onset type, the former being more common. However, malignant transformation is more common in adult onset type papillomatosis in spite of its relatively rarity[4,5]. Furthermore, it has been reported that most cases with malignant transformation are associated with smoking, irradiation and exposure to carcinogenic agents[5–8].

Correspondence: Takashi Hirano, Department of Surgery, Tokyo Medical College, 6-7-1 Nishishinjuku, Shinjuku-ku, Tokyo 160, Japan. Tel: 81-3-3342-6111; Fax: 81-3-3349-0326.

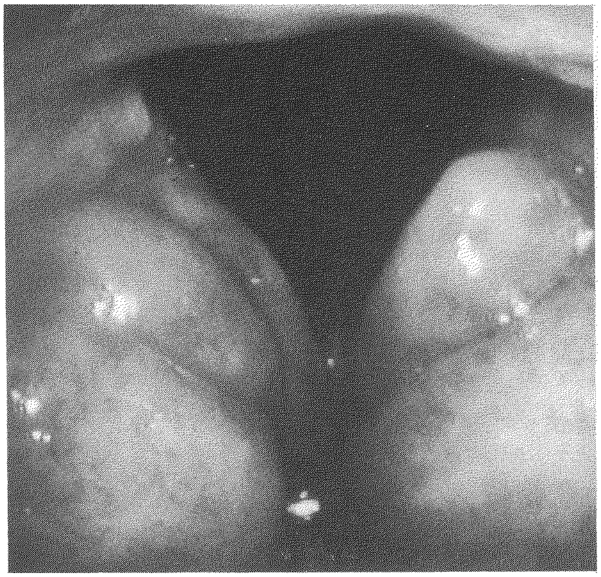

FIGURE 1 Laryngoscopic view of Laryngeal papilloma which resected in March 1993.

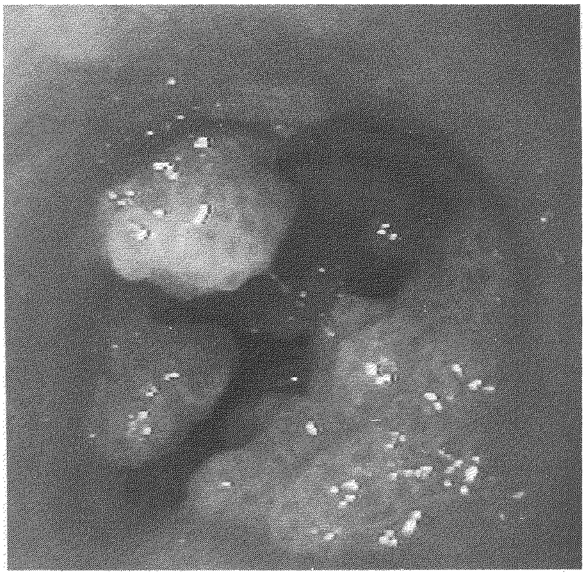

FIGURE 2 Bronchoscopic view of tracheal papillomatosis. Multiple polypoid tumors were observed in trachea.

The following case of adult onset type laryngotracheal papillomatosis had undergone endoscopic treatment for tracheal lesions using Nd-YAG laser. Two years after the initial treatments of laryngeal and tracheal papillomas, another solitary papilloma was discovered on the membranous portion of the trachea. We confirmed that this recurrent lesion was neither invasive papilloma nor squamous cell carcinoma by bronchoscopic ultrasound (US), helical computed tomography (CT) and tracheal biopsy. We discuss the usefulness of these methods in evaluating this recurrent lesion.

### CASE REPORT

A 62-year-old male, smoker presented at another hospital in March 1993. He had suffered from progressive hoarseness and cough over the previous two years. An otolaryngologist discovered laryngeal papilloma (Fig. 1), which was resected under laryngomicroscope. When he received follow-up examinations in August 1993, recurrence of laryngeal papilloma was discovered. At the same time tracheal involvement, was detected by bronchoscopy. After

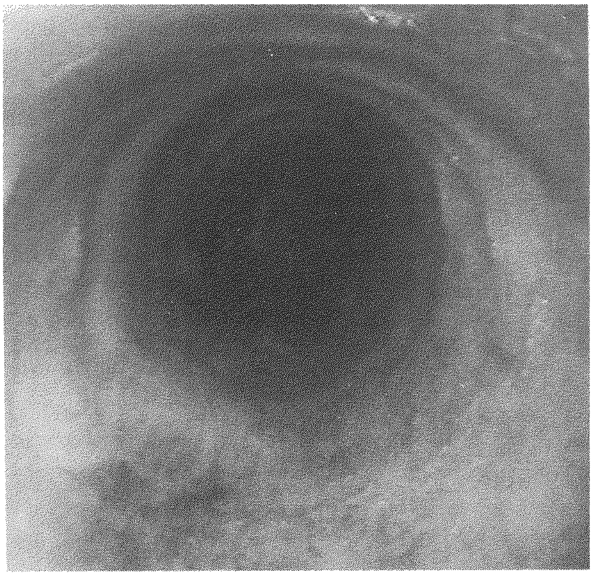

FIGURE 3 Bronchoscopic view of the trachea in March 1994. Complete remission was obtained by bronchoscopic vaporization in December 1993.

re-resection of the laryngeal papilloma, he was referred to our hospital for more detailed examinations and treatment.

On admission in November 1993 he complained of cough and sputum, with occasional wheezing audible on the anterior chest wall. However, the results of his physical examination were otherwise unremarkable, as were those of laboratory studies, including tumor markers (e.g CEA and SCC).

Bronchoscopy and bronchial biopsy revealed multiple polypoid tumors in the trachea, but none in the bronchi (Fig. 2). Histopathologically, papillary structures were composed of squamous epithelial cells. These epithelial cells were uniform in size with little evidence of pleomorphism or mitosis. Furthermore, histopathological specimens showed squamous epithelial cells with perinuclear clearing and binuclear (koilocytotic cells) suggestive of HPV infection. No evidence of malignant transformation was recognized. Based on conventional bronchoscopic, CT and histopathological findings we selected Nd-YAG laser vaporization for the tracheal papillomas. The laser beam was transmitted to the site of the mucosal lesions through a quartz optical fiber inserted through the working channel of a fiberopitic bronchoscope. We performed Nd-YAG laser irradiation three times, and total energy dosages of the irradiation were 5172 joules, 6329 joules and 2400 joules, respectively. The intervals between the three laser irradiation were approximately two weeks. Figure 3, taken after third laser treatment reveals no papillomas. The patient had no complications. After his discharge from our hospital he underwent bronchoscopic follow-up every four months.

In January 1996 we discovered a recurrent solitary papilloma on the membranous portion of the trachea (Fig. 4A). The margin of this smooth-surfaced lesion was distinct. We examined the invasive area of the papilloma lesion in the tracheal wall using bronchoscopic US (Olympus Optical Co., Tokyo, Japan, frequency: 20 MHz) and helical CT (5 mm collimation, reconstruction at 2 mm intervals for three dimensional images). The bronchoscopic US findings showed the intact epithelial and subepithelial layers (Fig. 4B). Therefore, we considered that the papilloma had not invaded the tracheal wall. Also, the helical CT findings corroborated no invasive lesion outside the tracheal wall and only exophytic growth (Fig. 4C). It was impossible to evaluate the multilayer structure of the tracheal wall using helical CT. Considering that the recurrent lesion was within the epithelial layer, Nd-YAG laser treatment was thought to be indicated. Total energy dosage for the recurrent lesion was 2120 joules.

### DISCUSSION

Respiratory papilloma is a relatively uncommon disease in adults while approximately 70% of this disease showing juvenile onset (at 10 years of age or less)[9]. The present case was one of adult onset type laryngotracheal papillomatosis in which bronchoscopic Nd-YAG laser vaporization was effective.

In this case, tracheal spread was discovered during follow-up for laryngeal papilloma. Furthermore, a recurrent solitary papilloma was discovered in the tracheal wall two years later. In general, respiratory papillomatosis is characterized by a high frequency of recurrence and spread into the distal respiratory tract. The rate of tracheal involvement by laryngeal papilloma has been reported to be 2–17%[5].

Before treating the recurrent tracheal solitary papilloma, we attempted to evaluate the extent and depth of the lesion in the tracheal wall as well as the histopathological findings using bronchoscopy (US and biopsy) and helical CT. Malignant transformation to squamous cell carcinoma is not unusual in smokers[6,8]. Also, there is a report that oat cell carcinoma can arise in tracheobronchial papilloma[10]. Furthermore, it has been reported that invasive tracheal papillomatosis without cellular atypia can grow through the tracheal cartilage[11]. Therefore, since this patient was a heavy smoker, it was necessary to evaluate the growth pattern and histopathological changes of the recurrent lesion before deciding on therapeutic strategy. Nd-YAG laser vaporization can be performed instead of surgical resection in certain benign tracheobronchial tumuors.

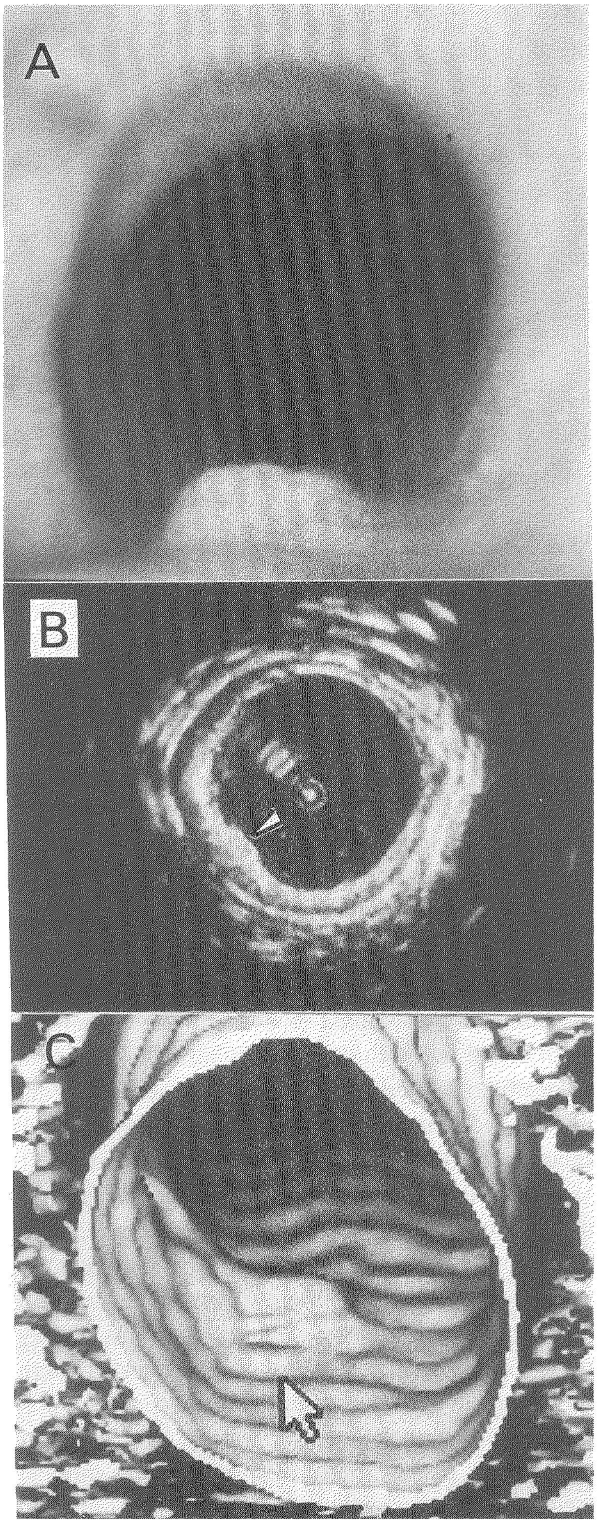

FIGURE 4A A recurrent solitary papilloma was discovered on the membranous portion in January 1996.

FIGURE 4B Bronchoscopic ultrasound findings of the recurrent papilloma. A small arrow shows the localization of the recurrent solitary papilloma. The border between epithelial layer and subepithelial layer is intact.

FIGURE 4C Helical computed tomography of the recurrent papilloma. Only exophytic growth can be observed.

However, confirmation that the lesion does not show invasive growth pattern is necessary. Conventional bronchoscopic US uses a 7.5 MHz US probe to evaluate beyond the tracheobronchial wall (e.g. lymph nodes and mediastinal tumor). Our bronchoscopic US with a 20 MHz US probe and a balloon, which was developed in collaboration with Olympus Optical Co., can analyze the multilayer structure of tracheobronchial wall. Bronchoscopic US findings suggested an intact border between the epithelial and subepithelial layers. We also checked beyond the tracheal wall using helical CT. Helical CT findings revealed only exophytic growth. Therefore, we again performed bronchoscopic resection under local anesthesia.

Although direct evidence of HPV infection was not established in this case, we diagnosed this case as laryngotracheal papillomatosis based only on the histopathological findings. Some squamous epithelial cells composing papillary structures exhihited perinuclear clearing and enlarged wrinkled nuclei consistent with koilocytes. Koilocytotic changes are often observed in viral induced lesions. It is very possible that this case was associated with HPV infection.

The degree of invasiveness of bronchoscopic vaporization under local anesthesia is less than surgical resection under general anesthesia (e.g. bronchoplasty, tracheoplasty). However, we have to consider the biological behavior of respiratory papillomatosis, such as the possibility of local recurrence, spread to the distal respiratory tract and malignant transformation. Evaluation of the extent and depth of papilloma lesions is needed to select the best treatment to prevent local recurrence. In recurrent lesions of respiratory papillomatosis it is especially important to evaluate the biological characteristics. We conclude that both bronchoscopic US and helical CT were very useful for deciding on therapeutic strategy.

### *Acknowledgments*

We would like to thank Professor J. Patrick Barron of the International Medical Communications Center of Tokyo Medical College for his review of this manucript.

### *References*

[1] Abramson, A.L., Steinberg, B.M. and Winkler, B.M. Laryngeal papillomatosis: clinical histopathologic and molecular studies. *Laryngoscope* 1987; **97**: 678–685.

[2] Corbitt, G., Zaroda, P., Arrand, J.R. *et al.* Human papillomavirus (HPV) genotypes associated with laryngeal papilloma. *J. Clin. Pathol.* 1988; **41**: 284–288.

[3] Terry, R.M., Lewis, F.A., Robertson, S. *et al.* Juvenile and adult laryngeal papillomata: classification by *in situ* hybridization for human papillomavirus. *Clin. Otlaryngol.* 1989; **14**: 135–139.

[4] Al Saleem, T., Peale, A.R. and Morris, C.M. Multiple papillomatosis of the lower respiratory tract. Clinical and pathologic study of eleven cases. *Cancer* 1968; **22**: 1173-1184.

[5] Mounts, P. and Shah, K.V. Respiratory papillomatosis: etiological relation to genital tract papillomaviruses. *Prog. Med. Virol.* 1984; **29**: 90–114.

[6] Helmuth, R.A., Strate, R.W. Squamous carcinoma of the lung in a nonirradiated, nonsmoking patient with juvenile laryngotracheal papillomatosis. *Am. J. Surg. Pathol.* 1987; **11**: 643–650.

[7] Solomon, D., Smith, R.R.L., Kashima, H.K., *et al.* Malignant transformation in nonirradiated recurrent respiratory papillomatosis. *Laryngoscope* 1985; **95**: 900–904.

[8] Guillou, L., Sahli, R., Chaubert, P. *et al.* Squamous cell carcinoma of the lung in a nonsmoking, nonirradiated patient with juvenile laryngotracheal papillomatosis. Evidence of human papillomavirus-11 DNA in both carcinoma and papillomas. *Am. J. Surg. Pathol.* 1991; **15**: 891–898.

[9] Weiss, M.D. and Kashima, H.K. Tracheal involvement in laryngeal papillomatosis. *Laryngoscope* 1983; **93**: 45–48.

[10] Blackman, F., Chung, H.R., McDonald, R.J. *et al.* Oat cell carcinoma with multiple tracheobronchial papillomatous tumors. *Chest* 1983; **5**: 817–819.

[11] Fechner, R.E. and Fitz-Hugh, G.S. Invasive tracheal papillomatosis. *Am. J. Surg. Pathol.* 1980; **4**: 79–86.

